# Psychotherapy for Physical Pain in Patients with Fibromyalgia: A Systematic Review

**DOI:** 10.1155/2020/3408052

**Published:** 2020-07-04

**Authors:** Lizzette Gómez-de-Regil, Damaris F. Estrella-Castillo

**Affiliations:** ^1^Hospital Regional de Alta Especialidad de la Península de Yucatán, Calle 7 No. 433 por 20 y 22, Fraccionamiento Altabrisa, Mérida 97130, Yucatán, Mexico; ^2^Universidad Autónoma de Yucatán, Facultad de Medicina, Licenciatura en Rehabilitación, Avenida Itzáes No. 498 x 59 y 59A, Colonia Centro, Mérida 97000, Yucatán, Mexico

## Abstract

**Objective:**

To provide a brief and comprehensive summary of the recent evidence from clinical trials testing psychotherapeutic interventions in patients with fibromyalgia with particular interest in their possible effect on physical pain.

**Methods:**

Bibliographical search was performed in PubMed, PsycInfo, Web of Science, Scopus, and Cochrane Library databases. Content of the manuscripts was studied to obtain, if available, the following information: year of publication, location of the research team, design, type of psychotherapeutic intervention tested, pain measures, and a brief description of the psychotherapy, groups, and outcomes regarding physical pain.

**Results:**

Initial search eliciting 475 citations got reduced to 13 relevant papers. Most research studies from Spain (*n* = 8) are randomized control trials (*n* = 10) and used guided imagery (*n* = 5) or cognitive behavioral therapy (*n* = 4). The Visual Analogue Scale (*n* = 4) and the Fibromyalgia Impact Questionnaire (*n* = 4) were the physical pain measures mostly used. Improvements on physical pain were reported on all studies with published results; nevertheless, only in five cases, differences were significant.

**Conclusions:**

Evidence on the effect of psychotherapy on physical pain in patients with FS was divergent; though most studies report a reduction in pain, this was not always lasting and/or significant. Diversity of the results might be due to the selected psychotherapeutic approaches, assessment tools, and other internal (e.g., personality traits, (sub)clinical psychiatric symptoms, and treatment adherence) and external (e.g., family environment and social support) variables worth to be considered in the future research.

## 1. Introduction

Fibromyalgia syndrome (FS) is a term used for identifying a physical condition characterized by chronic widespread pain perceived at musculoskeletal sites, often associated with poor sleep, fatigue, and depression [1,2]. A recent review on FS prevalence found values in the general population between 0.2 and 6.6%, in women between 2.4 and 6.8%, in urban areas between 0.7 and 11.4%, and in rural areas between 0.1 and 5.2% [3]. Despite its significant occurrence, its acknowledgment and delineation as a distinctive clinical disorder is relatively recent; in 1990, the American College of Rheumatology (ACR) provided the first classification criteria for FS [4] that was later updated [5] and modified [6]. In 1992, the World Health Organization officially coded FS for the first time, including it in the tenth Revision of the International Statistical Classification of Diseases and Related Health Problems (ICD-10).

FS is unlikely to resolve; less than 50% of patients achieve complete symptomatic relief, and less than 30% reach complete remission after 3 years of the diagnosis [1,7]. Thus, the current treatment alternatives mostly aim at managing pain and improving functionality and quality of life. Habitually, medical treatment prescribes antidepressant, antiepileptic, and analgesic drugs with modest benefits [8,9]. Nonpharmacologic therapies (e.g., cognitive behavioral therapy, psychoeducation, exercise, and vitamin D intake) and complementary therapies (e.g., tai chi, acupuncture, and manual or hydrotherapy) can provide further alleviation of physical and psychological symptoms [10,11].

Pain is the hallmark symptom in FS; yet, its cause is still unknown. Evidence suggests a multifactorial etiology in which central pain sensitization, disordered hypothalamic-pituitary-adrenal axis, vegetative tone anomaly, and sleep disturbance interplay as the FS pain triggers, mainly by the result of a reduced pain threshold [1,12]. Patients with FS experience continuous pain, not only physical, but also emotional. The use of antidepressant drugs and psychotherapy has shown favorable results in the amelioration of symptoms, suggesting a close link between the physical and the psychological dimensions of the syndrome [13].

Various studies have analyzed specific personality traits and/or psychiatric conditions (i.e., personality and/or mental disorders) that could be present in higher percentages in patients with FS. Studies report high levels of alexithymia (difficulty in recognizing and describing one's own emotions and feelings) and type D/distressed personality (elevated propensity to psychological distress due to a constant tendency to experience negative emotions across different life circumstances, called negative affectivity, and the restricted expression of emotions and behaviors in social interaction, called social inhibition) in patients with FS. Nevertheless, when depression is controlled, the results do not seem to differ from those of healthy controls [14]. Regarding psychopathology, the proportion of personality disorders appears far greater in patients with FS than in the general population, mainly obsessive-compulsive, avoidant, histrionic, and borderline personality disorders [15]. Moreover, compared with controls, patients with FS show a higher prevalence of mental disorders, particularly depression and anxiety, reported in 20–80% and 13–63.8% of cases, respectively [13].

From the field of psychology, researchers and professionals have worked intensively on the design, implementation, and testing of psychotherapeutic interventions in patients with FS [16]. Diverse psychological interventions have shown favorable results, from cognitive behavioral approaches [17,18] to psychoeducational [19] and mind and body therapies [20].

Given their particular health conditions, patients with FS may benefit from professional psychological support not only to deal with emotional issues directly or indirectly related to their illness, but also to manage physical pain, a frequent and disruptive symptom characterizing FS. This review presents a brief and comprehensive summary of the recent evidence from clinical trials testing psychotherapeutic interventions in patients with FS with particular interest in their possible effect on physical pain, a topic of potential interest to professionals working with this population and for patients themselves.

## 2. Methods

The review was performed according to the PRISMA statement (preferred reporting items for systematic reviews and meta-analyses [21]. A bibliographical search was performed in the PubMed, PsycInfo, Web of Science, Scopus, and Cochrane Library databases, February 20-21, 2020. The term “fibromyalgia” was entered in combination with “psychotherapy” and “pain,” restricted to title, abstract, and/or keywords when the option was available. Inclusion criteria were (1) research papers, (2) published in peer-reviewed journals, (3) available in English or Spanish, and (4) published during the last decade (2010 to February 2020). Exclusion criteria were (1) not original research (e.g., summaries, guidelines, reviews, and/or meta-analyses), (2) papers unrelated to the topic, (3) research not applied specifically to patients with FS and/or not using a psychotherapeutic intervention, and (4) physical pain was not included as an outcome measure. Publication relevance was verified based on the study objective. Citations for publications other than research articles (e.g., commentary, erratum, and book chapters) were excluded, and also articles reporting updates, (systematic) reviews, and/or meta-analyses. Abstracts were read to make a further cut of publications reporting research not clearly related to patients with FS and/or not focused on psychotherapeutic intervention. The full content of manuscripts was consulted to verify that physical pain was considered as an outcome measure of the interventional study. Papers reporting study protocols were included, considering that, even though they do not provide empirical evidence, they may offer a detailed description of the study design and the intervention, and their results are expected to be published afterwards. Once a final reference list was generated, the following information was obtained from each article: year of publication, location of the research team, design, type of psychotherapeutic intervention tested, pain measures, and a brief description of the psychotherapy, groups, and outcomes regarding physical pain. Both authors worked together through the procedure; discrepancies were minimal.

## 3. Results

**Figure 1:**
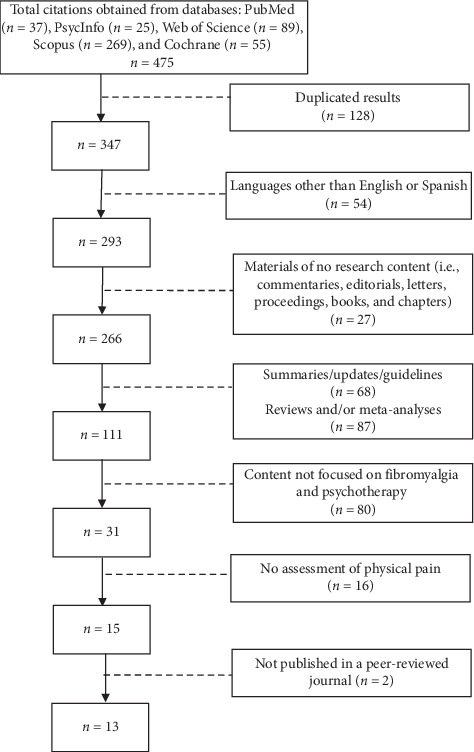
Study flow diagram.

The initial search from the five selected databases elicited 475 citations with 128 duplicates. Through the review of available abstracts, the list reduced to 31 research papers addressing psychotherapeutic interventions in patients with FS, and only 15 papers considered physical pain as an outcome measure. Then, two references of study protocols were excluded as they were not published in peer-reviewed journals. The final sample included 13 publications (Figure 1), with 11 original research manuscripts and two study protocols.

Table 1 summarizes the basic features of the publications. Most research comes from Spain (*n* = 8), followed by the United States (*n* = 2) and single contributions from Italy, the Netherlands, and Chile. Randomized control trials (RCT) are the most common design, 8 with two arms and 2 with three arms. Three studies used a single group design. Guided imagery was the most common psychotherapeutic intervention (*n* = 5), followed by cognitive behavioral therapy (CBT) (*n* = 4). The Visual Analogue Scale (VAS) (*n* = 4) and the specific items from the Fibromyalgia Impact Questionnaire (FIQ) (*n* = 4) were the physical pain measures mostly used. Others were the McGill Pain Inventory and the Brief Pain Inventory.

Table 2 presents brief information on interventions and their effect on physical pain. Most interventions were provided in weekly individual and/or group sessions, in small groups of 5 to 8 participants. Although only one research reported psychoeducation as an intervention, some others included psychoeducational topics as part of the program. The use of technology to provide psychotherapeutic treatment, for instance, recorded scripts and the implementation of a virtual reality environment, are mentioned. Regarding the effect of the psychotherapeutic intervention on physical pain, five publications reported a significant reduction in comparison with the control groups. One publication reported a significant improvement on physical pain, but not lasting. Five publications reported pain reduction but not at a significant level.  Table 3 summarizes the results on physical pain scores.

Additionally, both authors independently scored the studies using the scale for rating the quality of psychological trials for pain developed by Yates and colleagues [35]. Interrater reliability by the intraclass correlation coefficient (by the two-way mixed model and absolute agreement) was 0.95 (95% CI = 0.82–0.99) for the full scale, 0.65 (95% CI = 0.09–0.89) for treatment quality, and 0.97 (95% CI = 0.86–0.99) for methodological quality. Scores by raters are presented in Table 4.

## 4. Discussion

Fibromyalgia is not an uncommon syndrome, particularly among women [1,2]. On a daily basis, patients must face discomfort and pain in tender points throughout their bodies. Multidisciplinary treatment is recommended, including psychiatric/psychological services, as patients may experience mild to severe symptoms of depression and anxiety [36]. Furthermore, the use of antidepressants has shown improvements not only in the emotional state, but also in the self-perceived level of physical pain. This supports the idea of a possible connection between the physical and the psychological experiencing of pain in FS [13]. This review aimed at summarizing the recent evidence regarding psychotherapeutic interventions in patients with FS and their effect on physical pain.

The search found 13 publications on the topic; nevertheless, none of the psychotherapeutic interventions was specifically designed for the management of physical pain. Most studies followed a randomized control trial design that improves the reliability of the findings. In terms of outcomes, although in most cases physical pain was reduced, no effects of psychotherapeutic intervention could be established [22,23,26,28,29,32]. On the other hand, five studies found significant differences by the end of the treatment [27,30] and up to 3-month [24,31] and 12-month follow-ups [25]. Evidence is ambiguous; results could be influenced by the study design and the type of psychotherapeutic intervention, among other variables. It must be pointed out that physical pain was not the target outcome the interventions were designed for, and yet, in some cases, a significant effect was found. Further studies could focus on designing psychotherapeutic interventions for the amelioration of physical pain as a primary outcome, adapting contents and instruments to this objective.

Regarding the type of interventions, results show that guided imagery and CBT are the most common approaches. Imagery has been defined as “a mental function and a live experience that is a dynamic, quasireal, and psychophysiological process” [37]. Through imagery, initiated by the patient or guided by a therapist, the person creates and experiences an internal reality, regardless of the external stimuli, with the purpose of promoting adaptive changes in sensations, emotions, thoughts, or behaviors [38]. A recent meta-analysis by Zech and colleagues [39] found that guided imagery had a relevant benefit compared to controls on ≥50% pain relief at the end of therapy. Here, two studies found a significant effect on pain reduction at the end of the treatment [27,30] and one up to a 3-month follow-up [31], while other two studies could not establish a significant effect [29,32]. Guided imagery should be considered as a promising approach, not only for the available results in favor, but also due to the minimal economic cost of the intervention and that, once learned, the patient can continue practicing by him/herself. CBT assumes that negative emotions result from dysfunctional ideas framed by the person's system of beliefs. Therefore, CBT interventions guide the patients to identify those distorted beliefs that may influence the severity of the symptoms and to compromise to behavioral and cognitive changes, substituting dysfunctional schemes [40]. This review elicited three studies reporting on the use of CBT in patients with FS; nevertheless, none of them was targeted to reduce physical pain. Herrero and colleagues [23] proposed CBT for promoting positive emotions, showing significant increases in general mood state, positive emotions, motivation, and self-efficacy, but not a significant effect on physical pain. Lami and colleagues [24] developed a CBT program for insomnia in patients with FS; results showed that, regardless of sex, patients showed significant improvements in sleep quality and also in pain intensity. Lumley and colleagues [26] compared CBT and emotion awareness and expression therapy and found that they did not differ on pain severity although the latter led to a significantly lower widespread pain and a higher percentage of patients achieving 50% pain reduction. In a review, Bennet and Nelson [40] concluded that most studies found that CBT led to improvements in pain-related behavior and that sustained improvements were most evident when individualized CBT was used to treat patients with juvenile fibromyalgia. A CBT perspective might well improve the clinical outcome in FS, but not recommended as a single modality [40] but rather in a multidisciplinary program.

## 5. Conclusions

This systematic review of the recent evidence regarding the effect of psychotherapy on physical pain in patients with FS showed that results are divergent; though most reported a reduction in pain, this was not always lasting and/or significant. Diversity of the results might be due to the selected psychotherapeutic approaches, assessment tools, and other internal (e.g., personality traits, subclinical psychiatric symptoms, and treatment adherence) and external (e.g., family environment and social support) variables, and these are worth to be considered in the future research.

## Figures and Tables

**Table 1:** Identification features of publications.

Reference	Country	Design	Psychotherapeutic intervention	Pain measure
*Original research*				
[22]	Italy	Single-arm pre-post	Dance movement	Pain items from FIQ
[23]	Spain	Single-arm pre-post (pilot)	CBT for positive emotions	Numeric rating scale
[24]	Spain	Single-arm pre-post	CBT for insomnia	McGill Pain Questionnaire (short form)
				Pain items from FIQ
[25]	Spain	Two-arm RCT	Psychoeducation	Items from FIQ
[26]	USA	Three-arm RCT	Emotion awareness and expression therapy	Brief Pain Inventory
			CBT	
			Psychoeducation	
[27]	USA	Two-arm RCT	Guided imagery	Brief Pain Inventory (short form)
[28]	Spain	Three-arm RCT	Group problem solving	VAS
[29]	Spain	Two-arm RCT	Guided imagery	McGill Pain Questionnaire (long form)
				VAS
[30]	Spain	Two-arm RCT	Guided imagery	Pressure algometry
				Pain items from FIQ
[31]	Spain	Two-arm RCT	Group music and imagery method	Present Pain Intensity Scale of the McGill Pain Questionnaire
[32]	Netherlands	Two-arm RCT	Guided imagery	VAS

*Study protocol*				
[33]	Chile	Two-arm RCT	Behavioral activation	Composed Pain Intensity Index
[34]	Spain	Two-arm RCT	Personal construct therapy	VAS

FIQ: Fibromyalgia Impact Questionnaire; CBT: cognitive behavioral therapy; RCT: randomized control trial; VAS: Visual Analogue Scale; USA: United States of America.

**Table 2:** Brief description of the psychotherapeutic interventions and the results on physical pain.

Original research
[22] P*sychotherapeutic Intervention*. Dance movement psychotherapy (DMP). Ten weekly individual sessions. If needed, the program can be extended for up to 20 sessions. Each session begins with the therapist directing introductory warm-up exploring body connectivity, followed by the process of producing spontaneous/improvised movements. The session ends verbally discussing the possible meanings of the previously performed movements.
*Groups*. One group of 16 women
*Outcome*. After 10 weeks of intervention, the pain level reduced but not at a significant level
[23] *Psychotherapeutic Intervention*. Promotion of positive emotions through the use of a virtual reality environment. In groups of 6, participants were placed in front of a screen during 20 minutes, receiving simultaneously images, melodies, and narratives promoting relaxation, motivation, and positive emotions. Participants also received one session of psychoeducation about fibromyalgia and about the activity management component of the virtual reality device.
*Groups*. One group of 40 women
*Outcome*. There was a decrement from pretest to posttest on pain intensity, but it was not statistically significant
[24] *Psychotherapeutic Intervention*. Cognitive behavioral therapy for insomnia. The intervention was provided in groups of 5 to 7 participants, including only men or women. Participants received a protocol-based manual including psychoeducational information, exercises, and topics that would be discussed in the sessions and homework. There were 9 weekly sessions, lasting 90 minutes each.
*Groups*. One group of 28 participants, 15 women and 13 men
*Outcome*. Pain intensity reduced significantly through time (immediately after the intervention and three months later), with no interaction with sex
[25] *Psychotherapeutic Intervention*. *Psychoeducation*. The program included 5 sessions providing information about symptoms, course, comorbidities, potential causes, psychosocial factors, available treatments, benefits of regular exercise, and common barriers to behavioral change. There were also 4 sessions for autogenic training, a relaxation technique in which the person teaches the body to respond to commands to relax and control breathing, blood pressure, heart beat, and body temperature. Educational and autogenic training sessions were intercalated during 9 weeks, for 2-hour sessions, for a group of a maximum of 18.
*Groups*. Intervention group received usual care plus psychoeducation. Control group received only usual care (i.e., adjusted pharmacology and counseling about aerobic exercise). Each group included 108 participants.
*Outcome*. No difference between groups at baseline. At 12-month follow-up, patients who received psychoeducation reduced their score on pain, the control group increased its score, and differences were significant between groups.
[26] *Psychotherapeutic Intervention*. *Intervention Emotion Awareness and Expression Therapy*. This therapy aims at reducing amplification of central nervous system pain and sensory processes due to stress or conflicts, followed by emotional avoidance, by awareness, experiencing, and expression of more adaptive emotions. For each treatment option, patients met with therapist in groups of approximately 6, for eight 90-minute weekly sessions.
*Groups*. One group received emotional awareness and expression therapy (*n* = 79), a second group received cognitive behavioral therapy (*n* = 75), and a third group received fibromyalgia education (*n* = 76)
*Outcome*. Emotional awareness and expression therapy did not differ from education on pain severity. Compared to cognitive behavioral therapy, it did not differ significantly on pain severity, but it did have a higher percentage of patients achieving 50% pain reduction.
[27] *Psychotherapeutic Intervention*. *Guided Imagery*. Participants listened to three separate tracks of audio-recorded guided imagery scripts, instructed to use the tracks in 2-week increments and in consecutive order for the first 6 weeks; then to use the tracks in any order for follow-up weeks 7 through 10.
*Groups*. Control group receiving usual care and intervention group receiving usual care plus guided imagery. Each group with 36 participants.
*Outcome*. The intervention group showed significant decrease in pain severity from baseline to 6 weeks. From baseline to 10 weeks, the intervention group significantly reduced pain severity in comparison with the control group.
[28] *Psychotherapeutic Intervention*. Group problem-solving therapy. Three sessions at one-week intervals, and a fourth session at a one month interval after the third session.
*Groups*. Control group receiving cervical infiltration with botulinum toxin, the intervention group receiving group problem-solving therapy, and a third group receiving both therapies. Each group included 22 participants.
*Outcome*. No significant differences in pain before and after intervention, neither in total sample, nor in any of the three groups
[29] *Psychotherapeutic Intervention*. In the first session, the therapist guides the patient with an imagery exercise lasting 15 minutes. This is recorded in a CD and given to the patient with the instructions to listen to it at home at least 4 days during the first week and every day during the second week. The following week, the patient attends another session to practice relaxation, also recorded in a CD with the same previous instructions. In the following 4 weeks, the patient chooses any of the two CDs to listen as many days but only once daily.
*Groups*. Guided imagery group and a control group. Each group with 30 participants.
*Outcome*. At week 4, the intervention group reported statistically significantly lower levels of pain than the control group and a statistically significant effect on pain as measured by the daily VAS diary. At week 8, no significant differences were found for pain.
[30] *Psychotherapeutic Intervention*. *Guided Imagery*. Participants received three group sessions including guided imagery relaxation exercises, as well as group discussions and instructions on the use of the provided CDs. Participants were requested to do one exercise per day at least 4 times a week, during 8 weeks.
*Groups*. Guided imagery group (*n* = 28) and control group (*n* = 27)
*Outcome*. At week 4, the intervention group showed a significant reduction in pain at 5 out of 8 sensitive points. At week 8, these significant differences continued in 4 sensitive points. The control group showed no statistically significant differences in pain at sensitive points. No specific values on pain items from the FIQ are reported.
[31] *Psychotherapeutic Intervention*. *Group Music and Imagery*. During 12 weeks, participants attended two-hour weekly sessions in groups of 8. Sessions included relaxation, music listening, and spontaneous imagery.
*Groups*. Intervention group (*n* = 33) and control group (*n* = 26) condition
*Outcome*. Intervention group significantly decreased pain perception, up to three months after intervention
[32] *Psychotherapeutic Intervention*. *Guided Imagery*. A first group session included group discussion and theoretical background of guided imagery and received a CD with three guided imagery exercises to use at least one daily for the following four weeks. Then, in the second group session, the therapist led group discussion. Each session lasted 1.5 hour.
*Groups*. Intervention group (*n* = 32) and control group (*n* = 33)
*Outcome*. No effects on pain intensity of guided imagery could be established

Study protocol
[33] *Psychotherapeutic Intervention*. *Behavioral Activation*. Ten group sessions with 5 to 8 participants, over two months. Sessions focus on increasing activities associated with pleasure and reducing those that maintain or increase depression.
*Groups*. The control group will receive usual care for fibromyalgia with comorbid depression; the intervention group will receive in addition, behavioral activation therapy. Each group will include 45 participants.
[34] *Psychotherapeutic Intervention*. *Personal Construct Therapy and Cognitive Behavioral Therapy*. In each therapeutic condition, participants will attend up to eighteen 1-hr weekly sessions. After the end of treatment and during the following 3–5 months, participants will attend up to three 1-hr booster sessions.
*Groups*. One group will receive personal construct therapy and another group will receive cognitive behavioral therapy. Each group will include 45 participants.

**Table 3:** Summary of results from statistical analyses.

Reference	Mean scores (SD)	Comparison	*p*	*d*
[22]	Pre: NA	Pre vs post	0.5211	NA
	Post: NA			
	Difference pre vs post: −0.4 (1.9)			

[23]	Pre: 5.07 (2.03)	Pre vs post	0.086	0.12
	Post: 4.82 (2.24)			

[24]	*Men*			
	Pre: 7.40 (1.42)	Pre vs post	NA	1.69
	Post: 6.50 (1.96)	Post vs follow-up	NA	1.31
	Three-month follow-up: 6.55 (1.42)			
	*Women*			
	Pre: 7.30 (1.93)	Pre vs post	NA	0.09
	Post: 7.33 (1.88)	Post vs follow-up	NA	1.09
	Three-month follow-up: 6.93 (1.48)			

[25]	*Pre*			
	Intervention group: 7.37 (1.86)	NA	NA	NA
	Control group: 7.37 (2.10)			
	*Twelve-month follow-up*			
	Intervention group: 6.82 (2.34)	Intervention group vs control group	0.006	0.35
	Control group: 7.60 (2.08)			

[26]	*Pre*			
	EAET group: 5.34 (1.55)	NA	NA	NA
	CBT group: 5.35 (1.62)			
	Education group: 5.47 (1.74)			
	*Post*			
	EAET group: 4.48 (1.99)	EAET vs education	<0.01	−0.39
	CBT group: 4.69 (1.65)	EAET vs CBT	>0.05	−0.17
	Education group: 5.20 (1.68)	CBT vs education	>0.05	−0.23
	*Six-month follow-up*			
	EAET group: 4.40 (2.13)	EAET vs education	>0.05	−0.15
	CBT group: 4.82 (1.70)	EAET vs CBT	>0.05	−0.18
	Education group: 4.94 (1.96)	CBT vs education	>0.05	0.02

[27]	*Pre*			
	Intervention group: 5.3 (0.39)	NA	NA	NA
	Control group: 4.7 (0.37)			
	*Sixth week of intervention*			
	Intervention group: 4.7 (0.39)	Intervention group vs control group	0.03	NA
	Control group: 4.9 (0.37)			
	*Tenth week of intervention*			
	Intervention: 4.6 (0.39)	Intervention group vs control group	<0.01	NA
	Control: 5.1 (0.37)			

[28]	*Infiltration group*			
	Pre: 8.29 (1.67)	Pre vs post	>0.05	NA
	Post: 7.84 (1.83)			
	*GPST group*			
	Pre: 6.54 (1.85)	Pre vs post	>0.05	NA
	Post: 6.78 (2.01)			
	*GPST* *+* *infiltration group*			
	Pre: 7.35 (2.14)	Pre vs post	>0.05	NA
	Post: 8.13 (2.14)			

[29]	*Pre*	Intervention group vs control group		
	*MPQ sensory*			
	Intervention group: 17.4 (6.5)	MPQ sensory	0.221	NA
	Control group: 16.8 (10.1)			
	*MPQ affective*			
	Intervention group: 4.5 (5.8)	MPQ affective	0.086	NA
	Control group: 5.3 (5.3)			
	*MPQ evaluative*			
	Intervention group: 21.9 (9.4)	MPQ evaluative	0.897	NA
	Control group: 21.8 (10.5)			
	*Fourth week of intervention*	Intervention group vs control group		
	*MPQ sensory*			
	Intervention group: 16.8 (9.1)	MPQ sensory	0.039	NA
	Control group: 19.2 (10.2)			
	*MPQ affective*			
	Intervention group: 4.7 (6.4)	MPQ affective	0.044	NA
	Control group: 6.9 (8.8)			
	*MPQ evaluative*			
	Intervention group: 21.5 (7.5)	MPQ evaluative	0.041	NA
	Control group: 26.1 (9.7)			
	*Eight week (end) of intervention*	Intervention group vs control group		
	*MPQ sensory*			
	Intervention: 16.3 (9.1)	MPQ sensory	0.042	NA
	Control: 20.6 (10.6)			
	*MPQ affective*			
	Intervention: 4.2 (4.1)	MPQ affective	0.051	NA
	Control: 6.8 (3.9)			
	*MPQ evaluative*			
	Intervention: 20.5 (8.4)	MPQ evaluative	0.044	NA
	Control: 27.4 (9.32)			
	*Pre*	Intervention group vs control group	0.528	NA
	*VAS*			
	Intervention group: 7.66 (0.4)			
	Control group: 7.71 (0.8)			
	*Fourth week of intervention*	Intervention group vs control group	0.048	NA
	*VAS*			
	Intervention group: 5.89 (1.26)			
	Control group: 7.97 (1.12)			
	*Eight week (end) of intervention*	Intervention group vs control group	0.326	NA
	*VAS*			
	Intervention group: 8.05 (1.4)			
	Control group: 8.75 (1.47)			

[30]	*Baseline∗*			
	Intervention group/control group	Intervention group vs control group		
	Right occiput: 26/25		0.965	NA
	Left occiput: 23/22		0.947	NA
	Lower cervical (right side): 25/25		0.729	NA
	Lower cervical (left side): 24/21		0.481	NA
	Right trapezius muscle: 25/24		0.316	NA
	Left trapezius muscle: 25/20		0.117	NA
	Right supraspinatus muscle: 24/27		0.096	NA
	Left supraspinatus muscle: 24/27		0.096	NA
	Second right rib: 27/26		0.972	NA
	Second left rib: 29/25		0.072	NA
	Right lateral epicondyle: 25/26		0.422	NA
	Left lateral epicondyle: 26/25		0.189	NA
	Right gluteal muscle: 16/19		0.334	NA
	Left gluteal muscle: 13/22		0.063	NA
	Right greater trochanter: 15/11		0.678	NA
	Left greater trochanter: 22/13		0.696	NA
	Right knee: 19/19		0.510	NA
	Left knee: 20/16		0.364	NA
	*Four-week follow-up∗*			
	Intervention group/control group	Intervention group vs control group		
	Right occiput: 21/24		0.225	NA
	Left occiput: 20/21		0.620	NA
	Lower cervical (right side): 22/24		0.355	NA
	Lower cervical (left side): 15/22		0.034	NA
	Right trapezius muscle: 18/23		0.095	NA
	Left trapezius muscle: 16/21		0.121	NA
	Right supraspinatus muscle: 27/26		0.972	NA
	Left supraspinatus muscle: 26/26		0.676	NA
	Second right rib: 26/27		0.326	NA
	Second left rib: 25/28		0.042	NA
	Right lateral epicondyle: 25/26		0.422	NA
	Left lateral epicondyle: 24/26		0.253	NA
	Right gluteal muscle: 14/23		0.007	NA
	Left gluteal muscle: 12/21		0.010	NA
	Right greater trochanter: 10/12		0.525	NA
	Left greater trochanter: 7/13		0.080	NA
	Right knee: 20/20		0.463	NA
	Left knee: 20/20		0.334	NA
	*Eight-week follow-up∗*			
	Intervention group/control group	Intervention group vs control group		
	Right occiput: 24/24		0.577	NA
	Left occiput: 21/22		0.597	NA
	Lower cervical (right side): 24/25		0.487	NA
	Lower cervical (left side): 14/22		0.017	NA
	Right trapezius muscle: 19/24		0.376	NA
	Left trapezius muscle: 16/22		0.063	NA
	Right supraspinatus muscle: 24/27		0.096	NA
	Left supraspinatus muscle: 24/27		0.096	NA
	Second right rib: 25/27		0.422	NA
	Second left rib: 26/26		0.253	NA
	Right lateral epicondyle: 14/26		0.676	NA
	Left lateral epicondyle: 26/26		0.253	NA
	Right gluteal muscle: 14/21		0.039	NA
	Left gluteal muscle: 11/21		0.004	NA
	Right greater trochanter: 10/14		0.243	NA
	Left greater trochanter: 7/11		0.226	NA
	Right knee: 14/18		0.231	NA
	Left knee: 14/18		0.231	NA

[31]	*Pre*			
	Intervention group: 2.88 (1.02)	Intervention group vs control group	>0.05	NA
	Control group: 2.65 (0.98)			
	*Intervention group*			
	Pre: 2.86 (1.06)	Pre vs post	0.021	NA
	Post: 2.34 (1.20)	Pre vs follow-up	0.045	NA
	Three-month follow-up: 2.52 (1.06)			
	*Control group*			
	Pre: 2.65 (0.99)	Pre vs post	<0.05	NA
	Post: 2.65 (0.88)	Pre vs follow-up	<0.05	NA
	Three-month follow-up: 2.40 (1.05)			

[32]	*Pre*			
	Intervention group: 5.84 (NA)	Intervention group vs control group	<0.05	NA
	Control group: 5.88 (NA)			
	*Post*			
	Intervention group: 5.37 (NA)	Intervention group vs control group	<0.05	NA
	Control group: 5.33 (NA)			

CBT, cognitive behavioural therapy; EAET, emotion awareness and expression therapy; GPST, group problem-solving therapy; NA, not available; MPQ, McGill Pain Questionnaire; VAS, Visual Analogue Scale; ^∗^number of cases with pain.

**Table 4:** Assessment of the quality of the studies.

Reference	Treatment quality		Methodological quality		Overall quality	
	Rater 1	Rater 2	Rater 1	Rater 2	Rater 1	Rater 2
[22]	7	6	15	14	22	20
[23]	8	7	12	11	20	18
[24]	9	9	17	14	26	23
[25]	9	9	25	25	34	34
[26]	8	8	26	26	34	34
[27]	7	6	26	24	33	30
[28]	7	8	23	23	30	31
[29]	8	8	21	22	28	30
[30]	8	9	23	22	30	31
[31]	9	8	25	24	34	32
[32]	7	8	26	26	33	34
